# Deletion detection in SARS-CoV-2 genomes from COVID-19 patients: elimination of false positives

**DOI:** 10.1093/ve/veag003

**Published:** 2026-02-02

**Authors:** Nan Jiang, Colin N Dewey, John Yin

**Affiliations:** Wisconsin Institute for Discovery, University of Wisconsin-Madison, 330 N. Orchard St, Madison, WI 53715, United States; Department of Biostatistics and Medical Informatics, University of Wisconsin-Madison, 610 Walnut St, Madison, WI 53726, United States; Wisconsin Institute for Discovery, University of Wisconsin-Madison, 330 N. Orchard St, Madison, WI 53715, United States; Department of Chemical and Biological Engineering, University of Wisconsin-Madison, 1415 Engineering Dr, Madison, WI 53706, United States

**Keywords:** SARS-CoV-2, multiplex-PCR sequencing, genome deletion, false positives, bioinformatics filtering, ViReMa, STAR

## Abstract

Deletions are prevalent in the genomes of SARS-CoV-2 isolates from COVID-19 patients, but their roles in the severity, transmission, and persistence of disease are poorly understood. Millions of COVID-19 swab samples from patients have been sequenced and made available online, offering an unprecedented opportunity to study such deletions. Multiplex-PCR sequencing (amplicon-seq) has been the most widely used method for sequencing clinical COVID-19 samples. However, through experiments with negative control samples and existing bioinformatics methods, we find that this protocol introduces large numbers of false-positive deletions. These false positives commonly occur in short alignments, at low frequency and depth, and near primer binding sites used for whole-genome amplification. To address this issue, we developed a filtering strategy, validated with positive control samples containing a known deletion. Our strategy accurately detected the known deletion and removed >99% of false positives in Illumina short-read data from ARTIC amplicon sequencing protocols. This method, applied to public COVID-19 swab data, revealed that deletions occurring independently of transcription regulatory sequences were ~20-fold less common than previously reported; however, they remain more frequent in symptomatic patients. Our optimized approach should enhance the reliability of SARS-CoV-2 deletion characterization from surveillance studies. Finally, our approach may guide the development of bioinformatics pipelines for genome sequence analyses of other viruses.

## Introduction

COVID-19 is caused by severe acute respiratory syndrome coronavirus 2 (SARS-CoV-2), a single-stranded positive-sense RNA virus. Globally, as of January 2025, there have been 777 million confirmed cases of COVID-19, including 7 million deaths, reported to the WHO (WHO COVID-19 Dashboard 2025). Despite substantial public health interventions, the virus continues to evolve, necessitating ongoing genomic surveillance. Advances in sequencing technologies have enabled the collection of nearly 17 million SARS-CoV-2 genome sequences in Global Initiative on Sharing All Influenza Data (GISAID 2025). This wealth of data presents an unprecedented opportunity to study viral genome variants, including deletions, which can impact pathogenesis, transmission, and viral evolution (Vignuzzi and López 2019, Jeronimo et al. 2023).

Deletion variants of SARS-CoV-2 can be broadly classified as either replication-competent or defective. Replication-competent deletions are typically short (<50 nucleotides), often in-frame (3*n*), and may confer adaptive advantages (Young et al. 2020, Aguilar Rangel et al. 2023, Jeronimo et al. 2023). Notably, deletions, particularly in the spike gene, have been repeatedly acquired by several SARS-CoV-2 variants of concern, highlighting their potential role in immune evasion and host adaptation. However, the mechanisms by which these variant viruses are generated remain poorly understood. Defective deletion variants, commonly called ‘defective viral genomes’ (DVGs), lack at least one essential gene for growth and cannot reproduce in the absence of replication-competent virus. In general, DVGs can interfere with viral genome replication and packaging, trigger antiviral immunity, and facilitate persistent infection (Vignuzzi and López 2019). DVGs of SARS-CoV-2 have been detected in COVID-19 patients and virus cultures (Kim et al. 2020, Gribble et al. 2021, Wong et al. 2021, Zhou et al. 2023). Understanding the position, frequency, and distribution of deletion variants, both competent and defective, in clinical samples will likely be important for deciphering their roles in viral evolution and disease severity.

Given the scale of the available sequencing data, large-scale deletion analyses are now feasible. Most clinical SARS-CoV-2 sequencing relies on multiplex-Polymerase Chain Reaction (PCR) amplicon sequencing (amplicon-seq) for its sensitivity and cost-effectiveness. Amplicon-seq uses tiled PCR amplicons in two reaction pools to cover the viral genome (excluding the 3′ and 5′ ends) from cDNA generated by reverse transcription (Quick et al. 2017, Quick n.d.). The ARTIC and Midnight protocols are two commonly used amplicon-seq approaches, differing in amplicon length and primer design (Itokawa et al. 2020, Chiara et al. 2021, Constantinides et al. 2022).

Several bioinformatics tools, ViReMa (Routh and Johnson 2014), STAR (Wong et al. 2021), DI-tector (Beauclair et al. 2018), DVGFinder (Olmo-Uceda et al. 2022), and DVG-profiler (Bosma et al. 2019), detect deletions from short-read RNA-seq data. ViReMa and STAR are the most widely used, identifying split reads aligned to noncontiguous genomic regions, and here we use them simply as representative workflows rather than for tool benchmarking. However, their application to amplicon-seq data has not been systematically evaluated. Unlike RNA-seq, amplicon-seq relies on targeted PCR amplification, which can introduce artefacts (Zanini et al. 2017, Peccoud et al. 2018, Yan et al. 2021).

To address this gap, we systematically evaluated the outputs of standard bioinformatics pipelines using negative and positive control samples. Our analysis revealed that false-positive (FP) deletions are common in short alignments, low-frequency reads, and regions near primer-binding sites. Based on these findings, we developed a filtering strategy that reduces FPs while preserving true deletions. We validated this strategy using synthetic control samples containing a known deletion, demonstrating that our method detects true deletions with minimal FPs. Using our refined pipeline on publicly available COVID-19 swab sequencing data, we found that deletions occurring independently of transcription regulatory sequences (TRSs) are much less common than previously reported, but we confirmed their higher prevalence in symptomatic individuals.

This study provides an improved bioinformatics framework for reliable deletion detection in SARS-CoV-2 multiplex-PCR sequencing data. This approach can be readily adapted for studying other RNA viruses.

## Materials and methods

### Multiplex-PCR sequencing (amplicon-Seq) of the control samples

Synthetic deleted SARS-CoV-2 viral RNA (sDVR) was ordered from Trilink. Synthetic SARS-CoV-2 genomic RNA (vgRNA) was purchased from Twist Bioscience (#102024). The human reference RNA was obtained from Invitrogen (QS0639).

Control samples (Table 1) were reverse-transcribed using random primers and amplified via two multiplex-PCR reactions with ARTIC v3 or v5.3.2_400 primers (IDT), following the NEBNext® ARTIC SARS-CoV-2 protocol (Illumina E7650). Libraries were prepared using the QIAGEN FX DNA kit with fragmentation and sequenced on an Illumina NovaSeq X Plus platform (paired-end 150 bp reads).

**Table 1:** Control samples

Control sample components
Synthetic deleted SARS-CoV-2 viral RNA (sDVR) only
Synthetic SARS-CoV-2 genome RNA (vgRNA) only
Human reference RNA (hRNA) only
Human reference RNA and synthetic SARS-CoV-2 genome RNA (gRNA: hRNA = 1:300)^a^
Human reference RNA, sDVR, and synthetic SARS-CoV-2 genome RNA (gRNA:sDVR:hRNA = 1:1:300)^a^

^a^The ratios are calculated by mass. The expected frequency of the sDVR was ~0.91. The length of the sDVR is 3859 nt, whereas the full-length viral genome is 29 903 nt. The viral genomic RNA (vgRNA) obtained from Twist Bioscience (#102024) is labelled at a concentration of 1 × 10^6^ copies/μl, which corresponds to ~0.02 ng/μl. Our sDVR was supplied at 1 mg/ml. Based on mixing 0.4 ng of vgRNA (20 μl of a 1 × 10^6^ copies/μl stock) with 0.4 ng of sDVR (1 mg/ml stock, equivalent to 1.948 × 10^8^ copies). This ratio corresponds to 1.948 × 10^8^ ÷ (1.948 × 10^8^ + 2 × 10^7^) ≈ 0.91, with RNA copy numbers converted from mass using the NEBioCalculator. Thus, a 1:1 mass mixture of vgRNA and sDVR yields an expected deletion frequency of ~0.91.

### Initial prediction of deletions with ViReMa

Deletions (>5 nt) were predicted using ViReMa (v0.25) with modifications to the method of Gribble et al. (2021). Full command details are in the Supplementary Methods.

- Preprocessing: raw reads were processed to remove Illumina TruSeq adapters using Trimmomatic (v0.39). Reads shorter than 75 bp were discarded, and low-quality bases (*Q* score < 30) were trimmed. Paired reads were renamed to append ‘_1’ and ‘_2’ for R1 and R2 reads, respectively, and concatenated into a single FASTQ file.
- Alignment: the concatenated FASTQ files were aligned to the SARS-CoV-2 reference genome (NCBI NC_045512.2) using ViReMa (Viral Recombination Mapper, v0.25). We set ViReMa’s seed length to 20 to enable detection of our synthetic deletion, which is one nucleotide away from the ARTIC v3 primer 36_LEFT (10667-10 688).
- Frequency calculation: deletion coordinates were extracted from the alignment file, including information on deletion position, deletion depth, overhang length, and aligned nucleotide count. The alignment file was processed using Samtools (v1.10) to calculate nucleotide depth (coverage) at each position. Deletion frequency was calculated as the depth of deletion divided by the smaller depth at the donor or acceptor site.

### Initial prediction of deletions with STAR

Deletions (>5 nt) were also predicted using STAR (v2.7.3a) per Wong et al. (2021), with modifications.

- Preprocessing: raw paired-end reads were trimmed using Trim Galore (v0.4.3) via Cutadapt (v1.2.1). Reads shorter than 15 bp and low-quality bases (*Q* score < 30) were removed.
- Alignment: trimmed reads were aligned to the SARS-CoV-2 reference genome (NCBI NC_045512.2) using STAR (v2.7.3a) with Wong et al.’s command set.
- Frequency calculation: same as for ViReMa.

### Deletion summarization

Our final output file of deletion junctions contains the following information:

- chrom: reference name taken from FASTA file of mapped genome
- start: coordinate of the nucleotide right before the deletion (donor site)
- end: coordinate of the last deleted nucleotide (nucleotide right before acceptor site)
- depth: the number of reads containing the deletion
- depth_positive: the number of positive-strand reads containing the deletion
- depth_negative: the number of negative-strand reads containing the deletion
- n_fragments: the number of sequenced fragments (read pairs) containing the deletion
- max_left_overhang: the maximum number of mapped nucleotides before the deletion among reads containing the same deletion.
- max_right_overhang: the maximum number of mapped nucleotides after the deletion among reads containing the same deletion.
- max_aligned_length: the maximum number of mapped nucleotides among reads containing the same deletion
- start_cov: the coverage of the coordinate right before the deletion (donor site)
- end_cov: the coverage of the coordinate right after the deletion (acceptor site)
- MinCov: the lower coverage between the deletion’s start_cov versus end_cov
- Frequency: ‘depth’/‘MinCov’
- logFreq: log(‘Frequency’)
- deletion_length: length of the deletion (end–start)

### Filtering strategy

**Figure 1:**
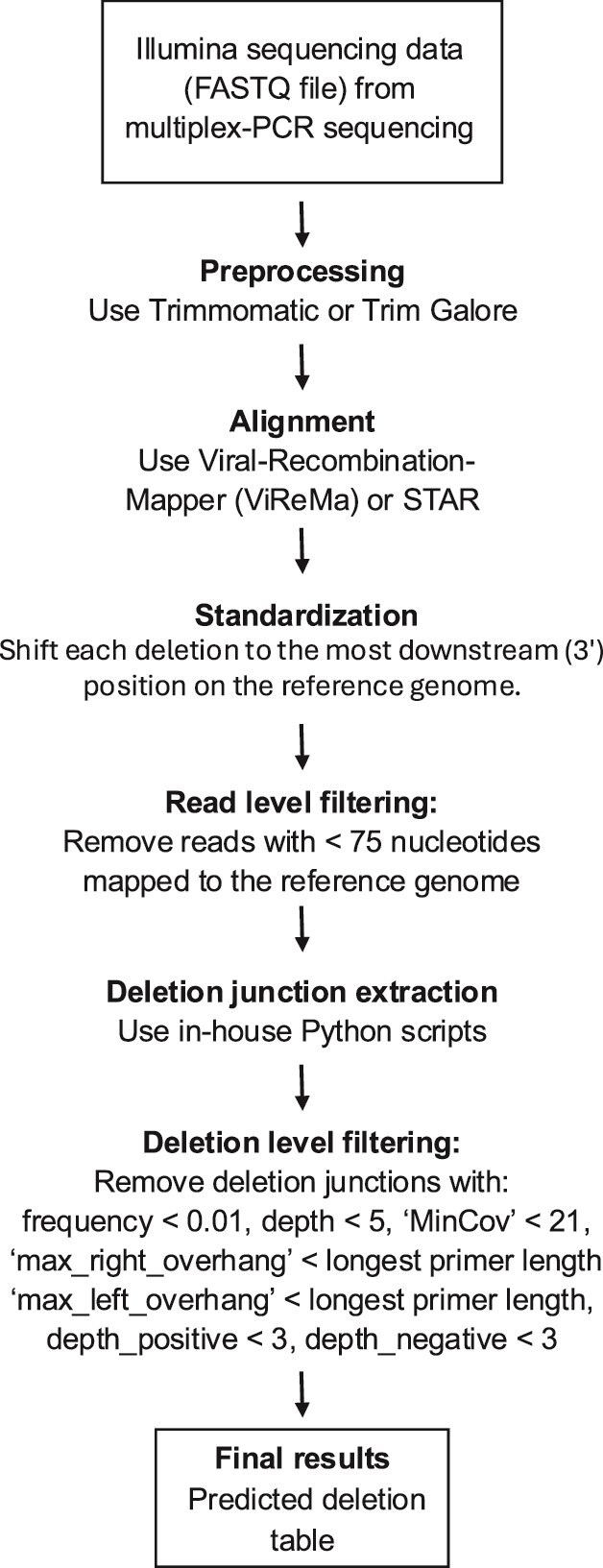
Optimized pipeline for identification of deletion junctions (>5 nt) in SARS-CoV-2 from multiplex-PCR Illumina sequencing.

The final optimized deletion identification pipeline is illustrated in Fig. 1. Full command details are in the Supplementary Methods.

### Identification of deletions associated with transcription regulatory sequences (TRS-associated) and DVGs (TRS-independent)

**Figure 2:**
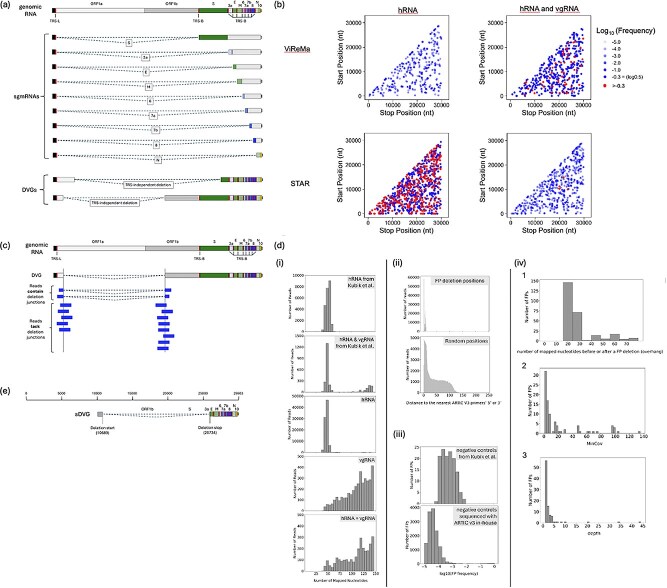
Origin, quantification, and elimination of false-positive (FP) deletions in amplicon sequencing. (a) Biological classes of SARS-CoV-2 RNA: viral genomic RNA, canonical subgenomic mRNA (sgmRNA), and defective viral genomes (DVGs). (b) False-positive deletion junctions detected in multiplex-PCR Illumina sequencing data by ViReMa and STAR, derived from human reference RNA (hRNA) and a mixture of hRNA and synthetic SARS-CoV-2 viral genomic RNA (vgRNA). Coverage summary of samples are in Supplementary Table S1. Deletion junctions are mapped according to their genomic position (5′ junction site, start position; 3′ junction site, stop position). The transparency represents their log10(frequency), so −2.0 has a frequency of 10^−2^ or 0.01. All the deletion junctions with frequency > 0.5 (log10(0.5) = −0.3) are labelled in red. (c) Calculation of deletion frequency. Deletion frequency is calculated as the number of reads supporting a given deletion junction divided by the lower of the coverage depths at the donor (start) and acceptor (stop) sites. Because detection requires amplification by primers flanking both sites, the lower coverage depth reflects the limiting primer pair and thus constrains detection sensitivity. In the example shown, the deletion frequency is 2/7 (≈0.29). (d) Diagnostic signatures of FP deletions. (i) Distribution of mapped nucleotides in FP-containing reads detected by ViReMa from hRNA and hRNA + vgRNA samples from Kubik et al., and negative control samples sequenced using ARTIC v3 primers. (ii) Distribution of distances from FP deletion start and end positions to the nearest ARTIC v3 primer ends (5′ or 3′) in hRNA samples sequenced with ARTIC v3 and analysed by ViReMa (upper panel) and distances from randomly selected genomic positions (matched in number to FP junctions) to the nearest primer ends (lower panel); mean distances are 3 nt for FP junctions and 41 nt for random positions. (iii) Distribution of FP deletion frequencies after removal of reads with <75 mapped nucleotides, shown for negative control datasets from Kubik et al. and for all ARTIC v3–sequenced negative controls analysed by ViReMa. (iv) Properties of remaining FP deletions (mapped nucleotides ≥ 75 nt, frequency ≥ 0.01) detected by ViReMa in ARTIC v3–sequenced negative controls (hRNA, vgRNA, and hRNA + vgRNA), including distributions of (1) minimum overhang length (min[max_left_overhang, max_right_overhang]), (2) minimum coverage at junction sites (MinCov), and (3) read support (depth). (e) Synthetic deleted viral genome RNA (sDVR) containing a deletion junction (10001–10689^26734–29903, inclusive) based on reference genome NC_045512.2. Owing to limitations of long RNA synthesis, the sDVR omits the first 10 000 nts of the SARS-CoV-2 genome.

We used the translate.pl script from Wong et al. to translate the viral RNA genome containing a single deletion (assuming there is only one deletion per genome). If the translated peptide sequence matched any of the peptide sequences derived from subgenomic RNAs (sgmRNAs) and the deletion’s start position was within the TRS-L (transcription regulatory sequence–leader) region, the deletion was classified as TRS-associated. All the other deletions were categorized as TRS-independent (DVG) deletions. Figure 2a illustrates SARS-CoV-2 TRS-associated deletions that result in sgmRNAs and TRS-independent deletions that create DVGs.

### Canonical subgenomic RNA deletion identification

Since deletions were standardized by shifting them as far downstream (3′ end) as possible, to ensure coordinate consistency, we standardized sgmRNA deletions by shifting them 3′-ward along the reference genome (NCBI NC_045512.2). A deletion was defined as canonical only if its coordinates exactly matched one of those in Supplementary Table S2.

### Data and code availability

The multiplex-PCR sequencing data for negative control samples from Kubik et al. (2021) were downloaded from NCBI (SRR13168458 and SRR13168423). Sequencing data for our control samples have been uploaded to NCBI (PRJNA1191883). COVID-19 swab sample sequencing data from Wong et al. were downloaded from NCBI (PRJNA690577). The code used for analysis is available on GitHub: https://github.com/NanJiang16/SARS-CoV-2_Deletion_detection_from_amplicon-seq.

## Results

### False-positive deletions are frequently detected in multiplex-PCR sequencing of negative controls

To assess the frequency of false positive (FP) deletions in amplicon-based sequencing, we analysed Illumina multiplex-PCR data from negative controls in Kubik et al. (2021) using pipelines from Gribble et al. (2021) and Wong et al. (2021). The main difference between these pipelines lies in their deletion detection software: Gribble et al. utilized ViReMa, while Wong et al. used STAR, both widely used for RNA virus analysis (Alnaji et al. 2019, Kim et al. 2020, Gribble et al. 2021, Wong et al. 2021, Martin et al. 2024). Kubik et al. utilized synthetic RNA reference genomes purchased from Twist Bioscience identical to the reference genome (NCBI NC_045512.2/Genbank ID MN908947.3, #102024), and Universal Human Reference RNA (Thermo Fisher Scientific #QS0639). We detected numerous FPs in negative controls analysed with either ViReMa or STAR (Fig. 2b). In a sample containing only human RNA (hRNA), ViReMa and STAR identified 691 and 1039 unique FPs, respectively; in a mixture of hRNA and synthetic viral RNA (vgRNA), they reported 379 and 551 FPs. To further evaluate the impact of sequencing depth and viral genome copy number, we analysed additional multiplex-PCR sequencing data from control samples reported by Kubik et al., using both methods. We found that FP counts were not correlated with viral genome copy number but appeared to be influenced by sequencing depth (Supplementary Table S3).

To quantify the frequency of a specific deletion, we calculated the ratio of deletion depth (reads supporting the deletion) to the smaller of the depths at the deletion’s donor and acceptor sites (Fig. 2c). This method addresses uneven coverage in multiplex-PCR sequencing, where the smaller depth, representing the less efficient primer pair, influences deletion detection. In the hRNA-only sample, the low depth of the viral genome led to consistently high FP frequencies, whereas the mixture exhibited a broader distribution of FP frequencies (Fig. 2b).

In addition to the publicly available sequencing data, we sequenced three further negative control samples using multiplex-PCR Illumina sequencing with the ARTIC v3 primer set: (i) vgRNA only, (ii) hRNA only, and (iii) a mixture of vgRNA and hRNA (vgRNA:hRNA = 1:300 by mass). The vgRNA and hRNA used were identical to those in Kubik et al. (2021). Consistent with the results from the public datasets, numerous FPs were found by both pipelines in all three negative control samples (Table 2). In the absence of vgRNA, the low depth of the viral genome led to consistently high FP frequencies.

**Table 2:** FPs in three negative control samples sequenced by multiplex-PCR Illumina sequencing using ARTIC v3 primer set before filtration

Control sample	vgRNA	hRNA	vgRNA and hRNA
Number of FPs predicted by ViReMa	7644	2124	6712
Number of FPs predicted by STAR	8345	1136	7697

### False-positive deletions often occur in short alignments and at low frequency

Analysis of the alignment files revealed that reads containing FPs could be stratified by the number of mapped nucleotides. In the hRNA sample, all the FP reads had <75 mapped nucleotides, whereas the control sample containing both hRNA and vgRNA exhibited FP reads predominantly shorter than 75 nucleotides or longer than 100 nucleotides (Fig. 2d(i)). The hRNA sample lacked viral RNA, so SARS-CoV-2 sequences should be absent. Any alignments in the hRNA sample likely originated from primers used for cDNA amplification. Using our knowledge of the ARTIC v3 primer design, we examined reads containing FPs in the samples we sequenced and found that FPs clustered around ARTIC v3 primers. The average distance between FP junctions and primer ends was 3 nt, far shorter than the random expectation of 41 nt (Fig. 2d(ii)). This finding suggests that most FPs were associated with ARTIC primers, potentially due to primer dimers (McCall et al. 2014), especially in samples without vgRNA, in which most FPs came from reads with <75 mapped nucleotides (roughly twice the length of the ARTIC v3 primers).

Based on this characterization, we stratified the reads into two groups: (1) reads with <75 mapped nucleotides and (2) reads with ≥75 mapped nucleotides. In Kubik et al.’s data, all the hRNA-only FPs fell into group 1. In vgRNA and hRNA mixtures, 70% of FPs were in group 1 and 30% in group 2. Most group 2 deletions occurred at frequencies < 0.01, consistent with the background error rate (~0.001) in amplicon sequencing (Fig. 2d(iii)). These trends were reproduced in our ARTIC v3-sequenced controls.

High-frequency FPs (≥0.01) in group 2 had additional hallmarks of artefacts: limited overhangs (<30 nt, the maximum length of ARTIC v3 primers), low coverage (MinCov ≤ 20), and minimal read support (depth < 5), indicating likely origins from primer sequences and poor amplification (Fig. 2d(iv)).

### Filtering eliminates most false positives while retaining known deletions

To reduce FPs while preserving true deletions, we applied a series of filters based on known FP characteristics. We excluded reads with <75 mapped nucleotides and retained only deletions with frequencies ≥ 0.01. While FPs often occur near primer sites, excluding all deletions within 5 nt of primers would eliminate ~13% of the genome. Instead, we used overhang length: deletions were retained only if both the max_right_overhang and max_left_overhang > 30 nt (the maximum ARTIC v3 primer length). Unlike read-level filters, this deletion-level approach ensures that a deletion interval is supported as long as some reads have longer overhangs, even if individual reads may have shorter overhangs.

We further filtered deletions by requiring ≥5 supporting reads (depth ≥ 5), both donor and acceptor (start and end) positions’ coverage depths > 20 (MinCov > 20), and representation on both strands (depth_positive and depth_negative > 2), as true RNA deletions should be detected in reads from both positive and negative strands after ARTIC-specific PCR amplification and library preparation. Figure 1 summarizes the filtering pipeline.

To validate this approach, we used a synthetic deleted SARS-CoV-2 viral genome RNA (sDVR) containing a known deletion (10001–10 689^26 734–29903, inclusive coordinates) (Fig. 2e) (Kim et al. 2020). Two types of positive control samples were prepared: sDVR only, and a mixture of vgRNA, sDVR, and hRNA (vgRNA:sDVR:hRNA = 1:1:300 by mass). With the 1:1 ratio of vgRNA to sDVR by mass, the expected frequency of the deletion was 0.91. Our filtering strategy eliminated most STAR-derived FPs and consistently detected the synthetic deletion, whereas ViReMa failed to call any deletions postfiltering (Table 3).

**Table 3:** FPs and synthetic deletion (SD) in positive control samples sequenced by multiplex-PCR Illumina sequencing using ARTIC v3 primer set before and after the filtration and standardization

Deletion detection tool	Positive control sample	sDVR	sDVR, vgRNA, and hRNA
ViReMa	Number of FPs before filtration	2637	5147
	Number of FPs after filtration	0	0
	Number of FPs after filtration and standardization	6	0
	Frequency of the SD before filtration	0.57	0.36
	Frequency of the SD after filtration	0.00	0.00
	Frequency of the SD after filtration and standardization	0.74	0.40
STAR	Number of FPs before filtration	3 940	5 573
	Number of FPs after filtration	14	3
	Number of FP after filtration and standardization	12	2
	Frequency of the SD before filtration	0.93	0.55
	Frequency of the SD after filtration	0.93	0.55
	Frequency of the SD after filtration and standardization	0.91	0.55

Closer inspection of ViReMa calls revealed near-identical junctions (10688^26733 and 10689^26734) on opposite strands, likely artefacts of ambiguous alignments near nucleotide repeats. Since ViReMa standardizes deletions in a strand-specific manner, this discrepancy prevented consolidation. We addressed this by developing a standardization process (standardize_alignments.py) for alignment files, ensuring each deletion was shifted as far downstream (3′ end) on the reference genome as possible, similar to the approach of Martin et al. (2024). This allowed ViReMa to recover the true deletion but introduced six FPs. STAR did not require standardization, as it inherently resolves strand discrepancies.

Currently, even with the latest alignment algorithms, it is challenging to predict deletion coordinates precisely when nucleotide repeats flank the junction. By combining filtration and standardization, we successfully detected the synthetic deletion in all the positive controls by both methods (Table 3) and eliminated all FPs in all the negative controls from both Kubik et al.’s and our sequencing data.

To test robustness across primer sets, we resequenced controls using ARTIC v5.3.2_400 (max primer length 34 nt). Our filters remained effective, removing nearly all FPs and detecting the known deletion with both STAR and ViReMa (Table 4). Manual inspection of the remaining FPs after filtration and standardization across all the samples revealed three main explainable classes: FPs near true-positive coordinates in the sDVR sample likely arising from synthetic RNA or PCR/sequencing errors; FPs whose breakpoints coincide (±1 nt) with primer ends consistent with primer-associated artefacts; and FPs associated with microhomology (see online supplementary material). Stricter filtering of deletions near primer ends could further reduce these FPs; however, this would reduce sensitivity to true deletions that happen to fall near primer ends, such as the synthetic deletion used in this work. Similarly, although microhomology (≥5 identical bases flanking breakpoints) could potentially be used for additional filtering, we chose not to because sequence similarity may also reflect genuine mechanisms underlying real defective viral genome formation (7).

**Table 4:** FPs and SD in control samples sequenced by multiplex-PCR Illumina sequencing using ARTIC v5.3.2_400 primer set before and after the filters and standardization

Deletion detection tool	Control sample	vgRNA	hRNA	vgRNA and hRNA	sDVR	sDVR, vgRNA and hRNA
ViReMa	Number of FPs before filtration and standardization	9297	3136	7804	3637	5486
	Number of FPs after filtration and standardization	2	0	0	4	0
	Frequency of the SD before filtration and standardization	0.00	0.00	0.00	0.79	0.43
	Frequency of the SD after filtration and standardization	0.00	0.00	0.00	0.86	0.52
STAR	Number of FPs before filtration and standardization	9507	1112	8328	4233	6647
	Number of FPs after filtration and standardization	3	0	0	8	0
	Frequency of the SD before filtration and standardization	0.00	0.00	0.00	0.94	0.59
	Frequency of the SD after filtration and standardization	0.00	0.00	0.00	0.94	0.59

**Figure 3:**
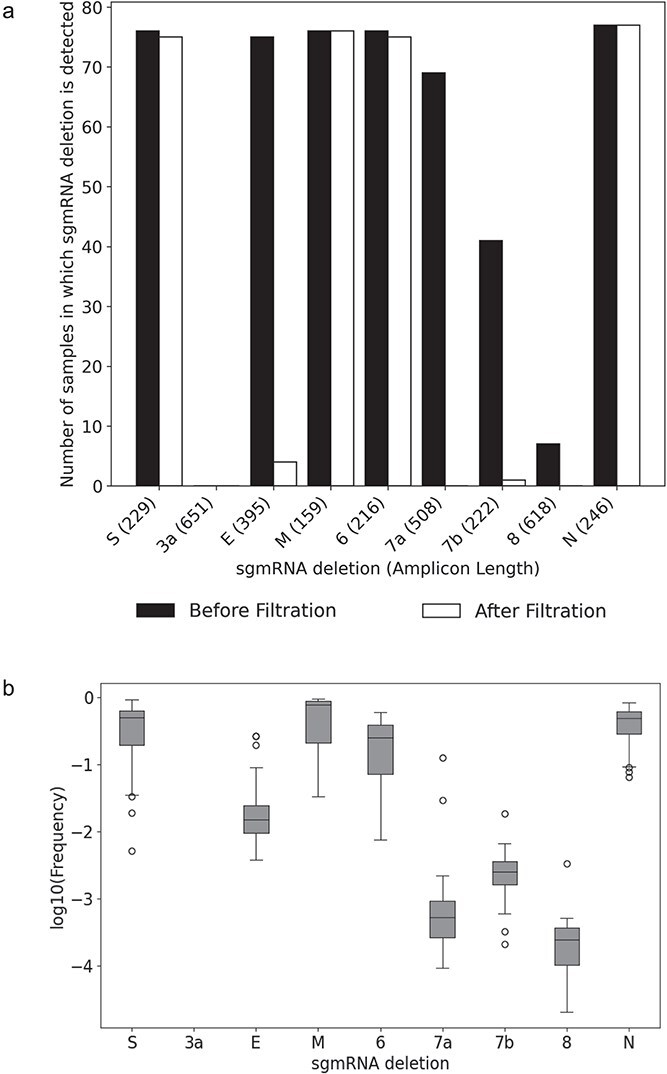
Detection of canonical SARS-CoV-2 subgenomic mRNA (TRS-dependent) deletions in COVID-19 swab samples. Canonical sgmRNA deletions were identified using STAR from multiplex-PCR Illumina sequencing data from 81 COVID-19 patient swabs (Wong et al.), before and after filtering. (a) Number of samples in which each sgmRNA deletion was detected. (b) Distribution of sgmRNA deletion frequencies among samples in which the deletion was detected prior to filtering.

To extend validation beyond a single large deletion, we also assessed the detection of canonical sgmRNA junctions, which are commonly found in patient swabs and represent a natural spectrum of biologically relevant deletion types of varying lengths and genomic contexts. We analysed data from 81 patient swab samples sequenced with ARTIC v3 primers from Wong et al. (2021). Of the six commonly detected sgmRNAs (S, E, M, 6, 7a, N) in patient samples, four (S, M, 6, N) were consistently retained after filtering (Fig. 3a). Detection depended not only on sgmRNA abundance but also on the length of the amplicon spanning the deletion. Deletions within longer amplicons (3a, E, 7a, 8) occurred at lower frequencies and were often missed or filtered out (Fig. 3b). Although the 7b amplicon is short, its low abundance also led to frequent exclusion. Although amplicon design imposes constraints on which sgRNAs can be confidently recovered, the subset detectable with strong support effectively broadens the range of validated deletion structures.

### Application of the filtering strategy to COVID-19 swab data

**Figure 4:**
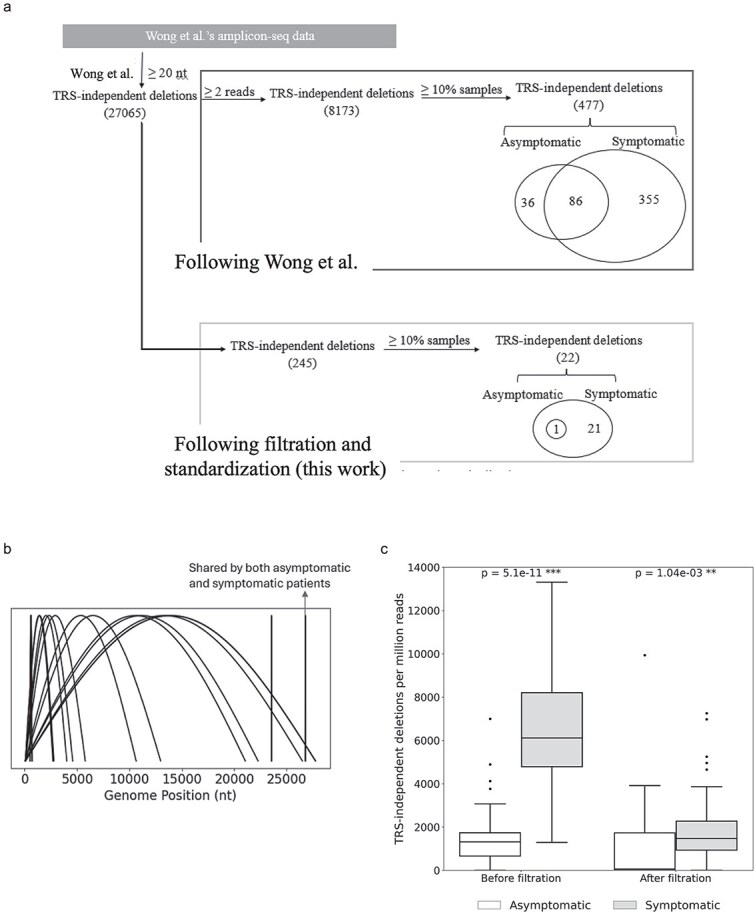
Filtering of COVID-19 swab sequencing data reduces detected TRS-independent deletions while preserving their higher prevalence in symptomatic hosts. (a) TRS-independent deletions inferred from multiplex-PCR Illumina sequencing data of swab specimens from symptomatic and asymptomatic patients. (b) Genomic distribution of TRS-independent deletions (*n* = 22) detected in symptomatic samples after standardization and filtering. (c) Distribution of normalized TRS-independent split-read counts (per million paired reads) before and after standardization and filtering, following the analysis framework of Wong et al. Statistical significance was assessed using the Mann–Whitney *U* test (center line, median; boxes, first and third quartiles; whiskers, 1.5× interquartile range).

Wong et al. sequenced 81 swab samples from patients (51 symptomatic, 30 asymptomatic) and analysed them with STAR (Wong et al. 2021). They found that symptomatic individuals harboured more and longer TRS-independent (DVG) deletions than asymptomatic individuals. To test the robustness of these findings against FPs, we initially repeated the published analysis and achieved similar results, to within 5%. Specifically, Wong et al. reported 501 deletions: 463 in symptomatic individuals, 126 in asymptomatic individuals, and 88 shared. Using their approach, we identified 477 deletions: 441 in symptomatic individuals, 122 in asymptomatic individuals, and 86 shared (Fig. 4a).

However, applying our filtering and standardization strategy to the same dataset reduced the total number of deletions by >20-fold. We detected 22 deletions present in ≥10% of symptomatic patients and 1 deletion present in ≥10% of asymptomatic patients; the latter (del 26 783–26 821 nt in the M gene) was the only event shared between both groups (Fig. 4a). Figure 4b summarizes the genomic locations of these variants (Supplementary Table S4). Many deletions are extremely large, removing essential regions of ORF1ab, in some cases, spike and downstream ORFs. All long deletions (>1000 nt) started within the first 100 nucleotides of the genome, suggesting that their formation may be associated with subgenomic RNA generation. A notable cluster of six short deletions falls within nsp1 (509–696 nt), overlapping a known hotspot where recurrent nsp1 deletions (e.g. del 500–532 nt) have been described and functionally linked to altered interferon antagonism (Lin et al. 2021). Although the exact breakpoints differ, their convergence on the same domain supports genuine biological variants rather than alignment artefacts. Three additional short deletions occur within the M gene (del 26 774–26 831 nt), a region shown to be highly conserved (Nguyen et al. 2021, Rogozin et al. 2023). This suggests that they may be replication-defective DVGs, which are worth further verification.

Furthermore, we compared normalized TRS-independent split-read counts (number of reads containing TRS-independent deletions) before and after filtering. While total split reads decreased postfiltering, symptomatic samples consistently exhibited a higher prevalence of deletion events, in agreement with the original findings (Fig. 4c). We also examined the impact of individual filters on the number of detected deletions (Table 5). The frequency filter (≥0.01) excluded the largest number of deletions, followed by the combined depth filter (depth ≥ 5, depth_positive > 2, and depth_negative > 2).

**Table 5:** Effects of different filters on the number of TRS-independent deletions

Process (without secondary alignments)	Number of deletions
Without filtration and standardization	27 065
Only with the number of aligned nucleotides on each read > 75	26 936
Only with standardization	27 071
**Only with frequency ≥ 0.01**	418
Only with depth ≥ 5 and depth_positive > 2 and depth_negative > 2	3676
Only with overhang length > 30	24 795
Only MinCov > 20	26 941

**Figure 5:**
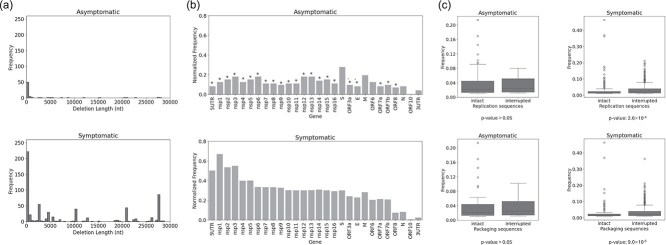
Structural and functional characteristics of TRS-independent deletions in COVID-19 patient samples. (a) Length distribution of all detected TRS-independent (DVG) deletions in asymptomatic and symptomatic COVID-19 patients. Histograms show deletion lengths aggregated across individuals; repeated deletions from different patients were counted repeatedly. Most deletions fall within the 5–500 nt range. (b) Gene-level distribution of TRS-independent deletions across the SARS-CoV-2 genome. Bars indicate the number of deletions affecting each viral gene, normalized by the total number of deletions; deletions spanning multiple genes were counted for each gene affected. Black stars denote statistically significant differences in normalized frequencies between patient groups (proportion test, *P* < .05). (c) Comparison of TRS-independent deletion frequencies that disrupt or retain known *cis*-acting replication and packaging sequences in symptomatic and asymptomatic patients. Deletion frequency was calculated as the depth of deletion divided by the smaller coverage depth at the donor or acceptor site. Statistical significance was assessed using two-sided Wilcoxon rank-sum tests (center line, median; boxes, first and third quartiles; whiskers, 1.5× interquartile range).

In addition to reproducing Wong et al’s analysis, we further characterized TRS-independent deletions detected from all the 81 patients. We revealed a broad deletion length distribution, with shorter deletions (5–500 nt) being far more prevalent. The average deletion length was longer in symptomatic patients (9377 nt) than in asymptomatic ones (3075 nt) (Table 6). Deletions > 500 nt were significantly less frequent in asymptomatic individuals (Fig. 5a). The distribution of deletions across the genome was also uneven, with some viral genes being disrupted more frequently than others (Fig. 5b). When deletions were categorized by their affected genes, deletions spanning multiple genes were counted for each gene involved. This analysis revealed distinct patterns of gene-specific deletion enrichment between patient groups. In the asymptomatic group, deletions affecting the *S* gene (encoding the spike protein) were more frequent, though this difference was not statistically significant. Overall, normalized deletion frequencies were lower in the asymptomatic group than in the symptomatic group across most genes, perhaps because deletions were generally shorter in asymptomatic group. These findings suggest that SARS-CoV-2 deletions may exhibit gene-specific patterns of enrichment that may vary with disease severity, possibly reflecting differential selective pressures or functional roles during viral replication and host interaction. To explore whether DVGs might still replicate or be packaged, we examined whether DVG (TRS-independent) deletions overlapped essential *cis*-acting elements required for replication and packaging.

**Table 6:** Average length of DVG deletions in different patient groups. Symptomatic (*n* = 51) and asymptomatic (*n* = 30) patients were from Wong et al.’s dataset

	Asymptomatic	Symptomatic	Two-sided Wilcoxon rank-sum test *P*-value
Average length of all DVG deletions	3 075	9 377	5.37 × 10^−13^
Average length of unique DVG deletions	2 847	6 841	1.01 × 10^−9^

Based on previous studies, replication sequences were defined as two regions spanning 1–634 nt and 29 534–29 903 nt (Girgis et al. 2022, Terasaki and Makino 2024), while packaging sequences encompassed three noncontiguous regions: 1–742, 15 071–16 451, and 29 278–29 903 nt (Terasaki et al. 2023, Chen et al. 2025). Packaging sequences therefore cover a broader portion of the genome and include all replication regions. DVGs retaining the full replication sequences could interfere with normal viral replication, while those retaining both replication and packaging sequences could interfere with normal virus replication and transmit between cells.

In patient-derived SARS-CoV-2 samples, deletions overlapping packaging sequences were more frequent than those disrupting replication sequences, consistent with their broader genomic coverage. Moreover, disruptions of packaging sequences occurred more frequently in symptomatic patients than in asymptomatic ones. Consequently, the proportion of deletions retaining intact packaging (and thus both replication and packaging) sequences was statistically lower in symptomatic individuals (Table 7). The frequency of DVG deletions within each patient group was also analysed and compared based on their disruption of the replication and packaging sequences. Deletions interrupting replication or packaging signals were found to be statistically more frequent than deletions keeping the sequences intact in the symptomatic group (Fig. 5c).

**Table 7:** Impact of DVG deletions on replication and packaging sequences across different patient groups. Summary of deletion counts and percentages highlighting interruptions of replication and packaging sequences in asymptomatic and symptomatic patient groups. Symptomatic (*n* = 51) and asymptomatic (*n* = 30) patients were from Wong et al.’s dataset

	Asymptomatic	Symptomatic	Proportion test *P*-value
Number of deletions that do not interrupt replication sequences	58	128	
Number of deletions that interrupt replication sequences	10	57	
Percentage of deletions that interrupt replication sequences	14.70	30.81	.016
Number of deletions that do not interrupt packaging sequences	51	92	
Number of deletions that interrupt packaging sequences	17	93	
Percentage of deletions that interrupt packaging sequences	25.00	50.27	.00056
Number of deletions that retain both replication and packaging sequences	51	92	
Percentage of deletions that retain both replication and packaging sequences	75.00	49.73	.00056

Together, these findings suggest that symptomatic patients may tend to harbour more DVGs lacking intact replication and packaging elements, implying that these DVGs may be less likely to be transmitted between cells.

## Discussion

The role of SARS-CoV-2 deletions in COVID-19 pathogenesis and viral evolution remains incompletely understood. The increasing availability of publicly shared sequencing data provides an unprecedented opportunity to investigate these deletions on a global scale. However, our study demonstrates that multiplex-PCR sequencing (amplicon-seq) introduces a significant number of FP deletions.

By systematically evaluating negative control samples, we identified common characteristics of FP deletions, including their prevalence in short alignments, proximity to primer-binding sites, and low frequency. These insights allowed us to refine deletion detection strategies through a multistep filtration process based on alignment length, deletion frequency, read depth, and overhang length.

Our filtering strategy significantly improves the reliability of deletion detection by minimizing FPs while preserving true deletions. Through validation using synthetic deletion-positive controls, we confirmed that our method retains true positive deletions while eliminating erroneous calls. When applied to patient swab datasets, our method revealed >20-fold fewer TRS-independent deletions than previously reported by Wong et al. Despite the overall reduction in detected deletions, our results support the previous observation that symptomatic COVID-19 patients exhibit a higher prevalence of TRS-independent deletions compared to asymptomatic individuals. The observed association between deletion patterns and disease presentation should be viewed as hypothesis-generating. The functional role of deletions or DVGs in clinical outcomes requires further mechanistic studies and larger population-level analyses. In addition, multiple technical and biological confounders may influence this association, including sampling time postinfection, RNA integrity, viral load, host immune response, and PCR amplification efficiency. Notably, sampling time postinfection has been reported to affect both DVG abundance and its apparent relationship with clinical outcomes (Felt et al. 2021; Penn et al. 2022).

While our optimized method effectively reduces FPs, several limitations remain in assessing the true positive rate. First, our analysis was constrained using a single synthetic deleted viral genome (sDVR) at only two concentrations. To improve this issue, we also assessed the ability of the method to detect sgmRNAs, which may be considered known deletions in swab samples. The method reliably detects sgmRNA deletions except for the sgmRNA deletions that required long amplicons and were found at low frequency. Nevertheless, performing sensitivity analysis and expanding validation experiments to include a broader range of deletion variants and concentrations would enhance the robustness of our approach while also allowing for a more comprehensive exploration of the inherent trade-off between false positive and true positive detections (precision vs recall) in methods like ours. Second, our frequency threshold of 0.01 prevents the detection of rare deletions, which may be relevant for studies on low-frequency viral populations or emerging variants. Third, we were unable to validate by wet-lab experiments that the deletions detected from Wong et al.’s dataset are “true biological” DVGs. However, the finding that few FPs were observed in our synthetic mixtures of sDVR, vgRNA, and hRNA, as well as in mixtures of vgRNA and hRNA, that closely mimic real biological samples suggests that these deletions represent genuine DVGs rather than artefacts that escaped the filtering pipeline. Fourth, while multiplex-PCR sequencing is the most widely used method for sequencing SARS-CoV-2 due to its high sensitivity and cost-effectiveness, it has intrinsic limitations in detecting deletions. Because this method relies on tiled amplicons for genome amplification in two reaction pools, deletions that begin before the 5′ end of the first primer in pool 2 or extend beyond the 3′ end of the last primer in pool 1 may not be detected. This limitation particularly affects the identification of sgmRNA deletions.

Additionally, multiplex-PCR sequencing cannot determine whether multiple distinct deletions originate from the same RNA molecule. Future work should explore complementary sequencing strategies, such as direct RNA sequencing, hybrid capture sequencing, or full-length cDNA sequencing, to overcome these limitations and the high input RNA requirement. Additionally, adapting our filtration approach to other RNA viruses could improve deletion detection across a broader range of viral genomic studies.

## Supplementary Material

Supplementary_Methods_Dec2025_veag003

Supplementary_Material_Annotated_False_Positives_veag003

Supplementary_Table_1_veag003

Supplementary_Table_2_veag003

Supplementary_Table_3_veag003

Supplementary_Table_4_veag003

## References

[ref1] Aguilar Rangel M, Dolan PT, Taguwa S et al. High-resolution mapping reveals the mechanism and contribution of genome insertions and deletions to RNA virus evolution. Proc Natl Acad Sci 2023;120:e2304667120. 10.1073/pnas.2304667120 37487061 PMC10400975

[ref2] Alnaji FG, Holmes JR, Rendon G et al. Sequencing framework for the sensitive detection and precise mapping of defective interfering particle-associated deletions across influenza A and B viruses. J Virol 2019;93:10–1128. 10.1128/jvi.00354-19 PMC6532088 30867305

[ref3] Beauclair G, Mura M, Combredet C et al. DI-tector: defective interfering viral genomes’ detector for next-generation sequencing data. RNA 2018;24:1285–96. 10.1261/rna.066910.118 30012569 PMC6140465

[ref4] Bosma TJ, Karagiannis K, Santana-Quintero L et al. Identification and quantification of defective virus genomes in high throughput sequencing data using DVG-profiler, a novel post-sequence alignment processing algorithm. PLoS One 2019;14:e0216944. 10.1371/journal.pone.0216944 31100083 PMC6524942

[ref5] Chen S-C, Xu C-T, Chang C-F et al. Characterization of the binding features between SARS-CoV-2 5′-proximal transcripts of genomic RNA and nucleocapsid proteins. RNA Biol 2025;22:1–16. 10.1080/15476286.2025.2471643 PMC11913385 40077853

[ref6] Chiara M, D’Erchia AM, Gissi C et al. Next generation sequencing of SARS-CoV-2 genomes: challenges, applications and opportunities. Brief Bioinform 2021;22:616–30. 10.1093/bib/bbaa297 33279989 PMC7799330

[ref7] Constantinides B, Webster H, Gentry J et al. Rapid turnaround multiplex sequencing of SARS-CoV-2: comparing tiling amplicon protocol performance. medRxiv 2021.12.28.21268461. 2021.12.28.21268461

[ref8] Felt SA, Sun Y, Jozwik A et al. Detection of respiratory syncytial virus defective genomes in nasal secretions is associated with distinct clinical outcomes. Nat Microbiol 2021;6:672–81. 10.1038/s41564-021-00882-3 33795879 PMC9098209

[ref9] Girgis S, Xu Z, Oikonomopoulos S et al. Evolution of naturally arising SARS-CoV-2 defective interfering particles. Commun Biol 2022;5:1–12. 36302891 10.1038/s42003-022-04058-5 PMC9610340

[ref10] GISAID . GISAID. 2025 https://gisaid.org/submission-tracker-global/ (30 January 2025, date last accessed).

[ref11] Gribble J, Stevens LJ, Agostini ML et al. The coronavirus proofreading exoribonuclease mediates extensive viral recombination. PLoS Pathog 2021;17:e1009226. 10.1371/journal.ppat.1009226 33465137 PMC7846108

[ref12] Itokawa K, Sekizuka T, Hashino M et al. Disentangling primer interactions improves SARS-CoV-2 genome sequencing by multiplex tiling PCR. PLoS One 2020;15:e0239403. 10.1371/journal.pone.0239403 32946527 PMC7500614

[ref13] Jeronimo PMC, Aksenen CF, Duarte IO et al. Evolutionary deletions within the SARS-CoV-2 genome as signature trends for virus fitness and adaptation. J Virol 2023;98:e01404–23. 10.1128/jvi.01404-23 38088350 PMC10804945

[ref14] Kim D, Lee J-Y, Yang J-S et al. The architecture of SARS-CoV-2 transcriptome. Cell 2020;181:914–921.e10. 10.1016/j.cell.2020.04.011 32330414 PMC7179501

[ref15] Kubik S, Marques AC, Xing X et al. Recommendations for accurate genotyping of SARS-CoV-2 using amplicon-based sequencing of clinical samples. Clin Microbiol Infect 2021;27:1036.e1–8. 10.1016/j.cmi.2021.03.029 PMC8016543 33813118

[ref16] Lin J, Tang C, Wei H et al. Genomic monitoring of SARS-CoV-2 uncovers an Nsp1 deletion variant that modulates type I interferon response. Cell Host Microbe 2021;29:489–502.e8. 10.1016/j.chom.2021.01.015 33548198 PMC7846228

[ref17] Martin MA, Berg N, Koelle K. Influenza A genomic diversity during human infections underscores the strength of genetic drift and the existence of tight transmission bottlenecks. Virus Evol 2024;10:veae042. 10.1093/ve/veae042 38883977 PMC11179161

[ref18] McCall CM, Mosier S, Thiess M et al. False positives in multiplex PCR-based next-generation sequencing have unique signatures. J Mol Diagn 2014;16:541–9. 10.1016/j.jmoldx.2014.06.001 25017478 PMC4188281

[ref19] Nguyen TT, Pathirana PN, Nguyen T et al. Genomic mutations and changes in protein secondary structure and solvent accessibility of SARS-CoV-2 (COVID-19 virus). Sci Rep 2021;11:3487. 33568759 10.1038/s41598-021-83105-3 PMC7876117

[ref20] Olmo-Uceda MJ, Muñoz-Sánchez JC, Lasso-Giraldo W et al. DVGfinder: a metasearch tool for identifying defective viral genomes in RNA-Seq data. Viruses 2022;14:1114. 35632855 10.3390/v14051114 PMC9144107

[ref21] Peccoud J, Lequime S, Moltini-Conclois I et al. A survey of virus recombination uncovers canonical features of artificial chimeras generated during deep sequencing library preparation. G3 Bethesda Md 2018;8:1129–38. 10.1534/g3.117.300468 29434031 PMC5873904

[ref22] Penn R, Tregoning JS, Flight KE et al. Levels of influenza A virus defective viral genomes determine pathogenesis in the BALB/c mouse model. J Virol 2022;96:e0117822. 10.1128/jvi.01178-22 36226985 PMC9645217

[ref23] Quick J . nCoV-2019 Sequencing Protocol V1. https://www.protocols.io/view/ncov-2019-sequencing-protocol-bbmuik6w (16 July 2024, date last accessed).

[ref24] Quick J, Grubaugh ND, Pullan ST et al. Multiplex PCR method for MinION and Illumina sequencing of Zika and other virus genomes directly from clinical samples. Nat Protoc 2017;12:1261–76. 10.1038/nprot.2017.066 28538739 PMC5902022

[ref25] Rogozin IB, Saura A, Bykova A et al. Deletions across the SARS-CoV-2 genome: molecular mechanisms and putative functional consequences of deletions in accessory genes. Microorganisms 2023;11:229. 10.3390/microorganisms11010229 PMC9862619 36677521

[ref26] Routh A, Johnson JE. Discovery of functional genomic motifs in viruses with ViReMa–a Virus Recombination Mapper–for analysis of next-generation sequencing data. Nucleic Acids Res 2014;42:e11. 10.1093/nar/gkt916 24137010 PMC3902915

[ref27] Terasaki K, Makino S. Requirement of the N-terminal region of nonstructural protein 1 in *cis* for SARS-CoV-2 defective RNA replication. J Virol 2024;98:e0090024. 10.1128/jvi.00900-24 39194239 PMC11406973

[ref28] Terasaki K, Narayanan K, Makino S. Identification of a 1.4-kb-long sequence located in the nsp12 and nsp13 coding regions of SARS-CoV-2 genomic RNA that mediates efficient viral RNA packaging. J Virol 2023;97:e0065923. 10.1128/jvi.00659-23 37367225 PMC10373556

[ref29] Vignuzzi M, López CB. Defective viral genomes are key drivers of the virus–host interaction. Nat Microbiol 2019;4:1075–87. 10.1038/s41564-019-0465-y 31160826 PMC7097797

[ref30] World Health Organization (WHO). WHO COVID-19 Dashboard . 2025. https://data.who.int/dashboards/covid19 (30 January 2025, date last accessed).

[ref31] Wong CH, Ngan CY, Goldfeder RL et al. Reduced subgenomic RNA expression is a molecular indicator of asymptomatic SARS-CoV-2 infection. Commun Med 2021;1:1–12. 35602196 10.1038/s43856-021-00034-y PMC9053197

[ref32] Yan B, Chakravorty S, Mirabelli C et al. Host-virus chimeric events in SARS-CoV-2-infected cells are infrequent and artifactual. J Virol 2021;95:e0029421. 10.1128/JVI.00294-21 33980601 PMC8274596

[ref33] Young BE, Fong S-W, Chan Y-H et al. Effects of a major deletion in the SARS-CoV-2 genome on the severity of infection and the inflammatory response: an observational cohort study. Lancet 2020;396:603–11. 32822564 10.1016/S0140-6736(20)31757-8 PMC7434477

[ref34] Zanini F, Brodin J, Albert J et al. Error rates, PCR recombination, and sampling depth in HIV-1 whole genome deep sequencing. Virus Res 2017;239:106–14. 10.1016/j.virusres.2016.12.009 28039047

[ref35] Zhou T, Gilliam NJ, Li S et al. Generation and functional analysis of defective viral genomes during SARS-CoV-2 infection. Mbio 2023;14:e00250-23. 2022.09.22.509123 10.1128/mbio.00250-23 PMC10294654 37074178

