# Supplementary material for: Deletion detection in SARS-CoV-2 genomes from COVID-19 patients: elimination of false positives
Source: Virus Evol. 2026 Feb 2;12(1):veag003. doi: 10.1093/ve/veag003 (PMC12900060; doi:10.1093/ve/veag003)
Supplement: Supplementary_Table_1_veag003 [file supplementary_table_1_veag003.docx]

Supplementary Table 1. **Coverage summary of samples in Figure 2b.**

| sample | mean_coverage | fraction_>5 |
| --- | --- | --- |
| STAR_hRNA | 346.272013 | 0.489683 |
| STAR_hRNA_vgRNA | 6306.269204 | 0.98552 |
| ViReMa_hRNA | 853.973581 | 0.602816 |
| ViReMa_hRNA_vgRNA | 6262.477544 | 0.987192 |
