# Supplementary material for: Deletion detection in SARS-CoV-2 genomes from COVID-19 patients: elimination of false positives
Source: Virus Evol. 2026 Feb 2;12(1):veag003. doi: 10.1093/ve/veag003 (PMC12900060; doi:10.1093/ve/veag003)
Supplement: Supplementary_Table_2_veag003 [file supplementary_table_2_veag003.docx]

Supplementary Table 2. **FPs detected in control samples with different copy numbers of viral genomes before filtration with data from Kubik et al.** (SRR13168406, SRR13168373, SRR13168478, SRR13168434, SRR13168429)

| Copy number of viral genomes | 10 | 100 | 1000 | 10000 | 100000 |
| --- | --- | --- | --- | --- | --- |
| Number of FP predicted by ViReMa | 597 | 477 | 549 | 445 | 1355 |
| Number of FP predicted by STAR | 1270 | 1103 | 1196 | 829 | 1354 |
| Sequencing depth | 1.8M | 1.2M | 1.9M | 1.2M | 2.4M |
