# Supplementary material for: Deletion detection in SARS-CoV-2 genomes from COVID-19 patients: elimination of false positives
Source: Virus Evol. 2026 Feb 2;12(1):veag003. doi: 10.1093/ve/veag003 (PMC12900060; doi:10.1093/ve/veag003)
Supplement: Supplementary_Table_4_veag003 [file supplementary_table_4_veag003.docx]

Supplementary Table 4. **Coordinates of deletion junctions in Figure 4B.** Deletion 26783-26821 was shared by both asymptomatic and symptomatic patients

| **Start** | **End** | **Main region(s) impacted**  **(Wuhan-Hu-1)** |
| --- | --- | --- |
| 63 | 5785 | 5′UTR; nsp1–nsp2; nsp3 |
| 70 | 2794 | 5′UTR; nsp1–nsp2; nsp3 |
| 70 | 27761 | 5′UTR; all nsps (nsp1–nsp16); Spike (S) ; ORF3a; Envelope (E); Membrane (M); ORF6; ORF7a; ORF7b |
| 71 | 2692 | 5′UTR; nsp1; nsp2 |
| 71 | 22278 | 5′UTR; all nsps (nsp1–nsp16); S |
| 71 | 4022 | 5′UTR; nsp1–nsp2; nsp3 |
| 72 | 10643 | 5′UTR; nsp1–nsp4; 3CLpro / nsp5 |
| 73 | 4585 | 5′UTR; nsp1–nsp2; nsp3 |
| 75 | 21059 | 5′UTR; all nsps (nsp1–nsp15 plus nsp16) |
| 76 | 12976 | 5′UTR; nsps 1–8; nsp9 |
| 78 | 27769 | 5′UTR; all nsps (nsp1–nsp16); S; ORF3a; E; M; ORF6; ORF7a; ORF7b |
| 91 | 26507 | 5′UTR; all nsps (nsp1–nsp16); S; ORF3a; E (stops just upstream of M) |
| 509 | 686 | internal nsp1 deletion |
| 510 | 696 | internal nsp1 deletion |
| 517 | 695 | internal nsp1 deletion |
| 518 | 686 | internal nsp1 deletion |
| 518 | 695 | internal nsp1 deletion |
| 520 | 689 | internal nsp1 deletion |
| 23554 | 23583 | internal S gene deletion |
| 26774 | 26831 | internal M gene deletion |
| 26783 | 26821 | internal M gene deletion |
| 26786 | 26821 | internal M gene deletion |
